# AgRP neurons shape the sperm small RNA payload

**DOI:** 10.1038/s41598-025-91391-4

**Published:** 2025-02-28

**Authors:** Iasim Tahiri, Sergio R. Llana, Francisco Díaz-Castro, Marc Claret, Arnaud Obri

**Affiliations:** 1 Neuronal Control of Metabolism, Institut d’Investigacions Biomèdiques August Pi i Sunyer, Rosselló 149-153, 5th floor, Barcelona, 08036 Spain; 2 Doctoral Program in Biomedicine, Universitat de Barcelona, Barcelona, Spain; 3 CIBER de Diabetes y Enfermedades Metabólicas Asociadas (CIBERDEM), Barcelona, Spain; 4 School of Medicine, Universitat de Barcelona, Barcelona, Spain; 5 Physiology Department, Biological Science Faculty, Pontificia Universidad Católica de Chile, Santiago, Chile

**Keywords:** Molecular biology, Neuroscience, Epigenetics, Metabolism

## Abstract

Paternal dietary patterns and obesity can affect offspring through epigenetic signals in sperm RNA. By activating hypothalamic Agouti-related peptide (AgRP) neurons in mice to simulate obesity’s effects, we observed changes in the small noncoding RNA payload of sperm. These alterations, particularly in transfer RNA-derived small RNAs (tsRNAs), are similar to those induced by short-term high-fat diets, suggesting a common upstream regulatory mechanism involving AgRP neurons that affects metabolic epigenetic inheritance.

**Supplementary Information:**

The online version contains supplementary material available at 10.1038/s41598-025-91391-4.

## Introduction

Paternal metabolic state and dietary patterns affect the progeny through a cascade of epigenetic signals. These modifications, transported by the sperm RNA payload, are now recognized as vectors transmitting nuanced information that shapes offspring phenotypes^1–4^. Recent studies have shown that transfer RNA-derived small RNAs (tsRNAs) in sperm are important mediators of epigenetic inheritance, particularly influencing the metabolic pathways that predispose offspring to obesity^4–9^. The mechanisms underlying sperm RNA modifications are complex. Sperm RNA content can be altered through several established pathways: direct environmental effects on developing sperm cells in the testes, RNA transfer from epididymal cells via epididymosomes, mitochondrial dysfunction, and RNA modifications and processing^4,5,10–12^.

This complexity of RNA modification pathways suggests multiple potential points where metabolic signals could influence the sperm RNA payload. In this context, hypothalamic Agouti-related peptide (AgRP) neurons emerge as particularly interesting candidates for investigation, as these neurons are central players in the orchestration of energy balance and are known to modify their activity in response to excessive weight gain, reflecting an intricate dialogue between nutritional status and neuroendocrine function^13^. The influence of AgRP neurons extends beyond metabolic regulation, as their activation triggers a complex network of downstream effects that impact various physiological processes such as reproductive function and cognitive function among others^14–16^. While the transformative power of the sperm RNA payload is acknowledged, particularly concerning metabolic traits, the precise signals that sculpt these epigenetic landscapes remain to be discovered.

**Fig. 1:**
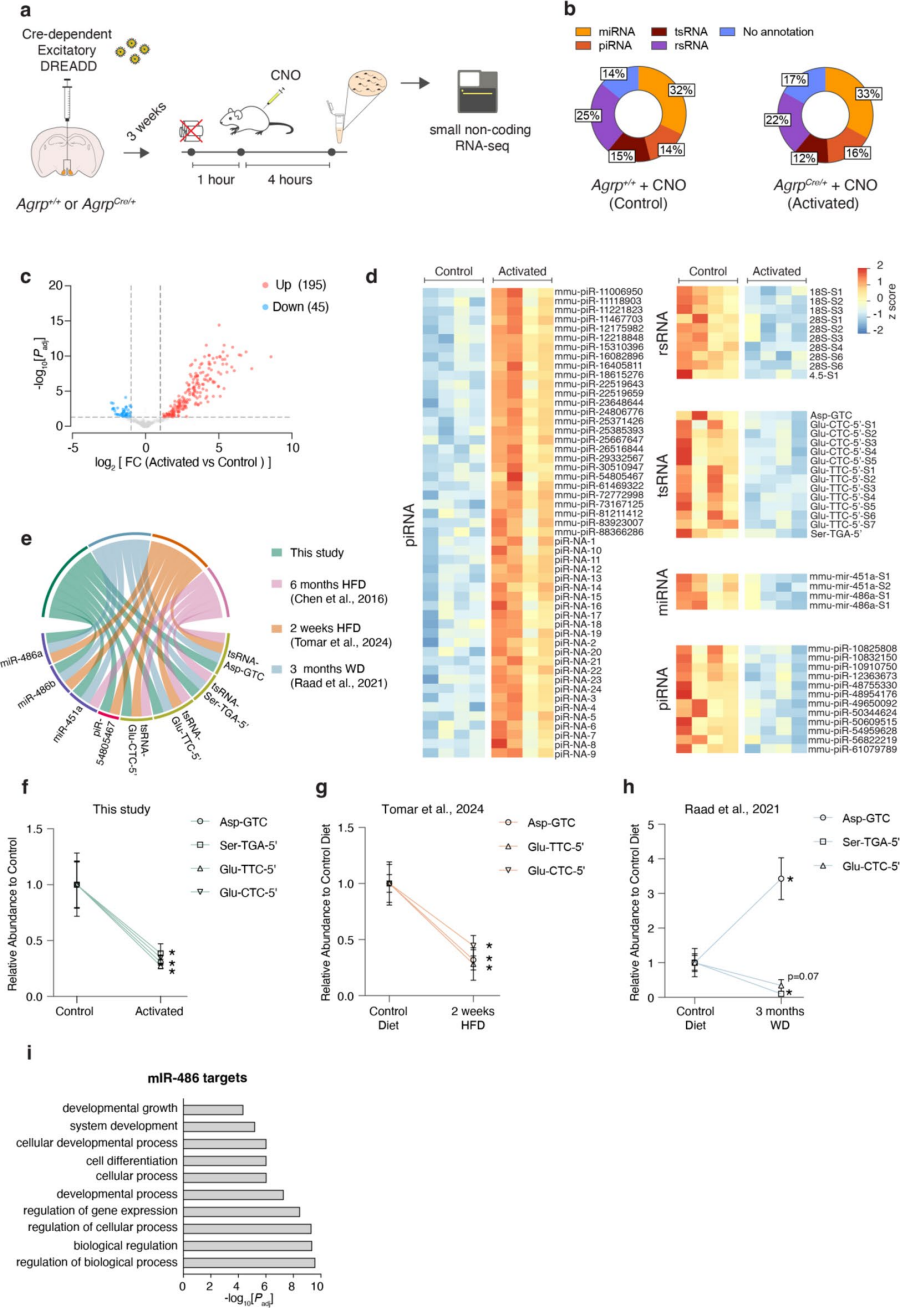
Activation of hypothalamic AgRP neurons leads to alteration on the sperm RNA payload. (**a**) Experimental design of this study. (**b**) Distribution of sncRNA biotypes in cauda spermatozoa from control (*Agrp*^*+/+*^ + CNO) and activated (*Agrp*^*Cre/+*^ + CNO) mice. *n* = 4 per group. microRNAs (miRNAs), PIWI-interacting RNAs (piRNAs), transfer RNA-derived small RNAs (tsRNAs) and ribosomal small RNAs (rsRNAs). (**c**) Volcano plot representation of differentially expressed small non coding RNAs ( sncRNAs) fragments (false discovery rate < 0.05 and log2[fold change (FC)] > |1|). *n* = 4 per group. (**d**) Heat map representation of differentially expressed piRNA, miRNA, rsRNA and tsRNA fragment levels in cauda spermatozoa from control and activated mice. *n* = 4 per group. (**e**) Chord diagram illustrating the overlap of significantly (false discovery rate < 0.05) altered sncRNAs between our study and previous studies examining the impact of high-fat diet (HFD) or Western diet (WD) feeding on sperm RNA payload composition. (**f**) Relative abundance of tsRNAs (Asp-GTC, Ser-TGA-5’, Glu-TTC-5’, Glu-CTC-5’) differentially expressed fragments in control (*n* = 4) and AgRP-activated (*n* = 4) groups (this study). (**g**) Relative abundance of tsRNAs (Asp-GTC, Glu-TTC-5’, Glu-CTC-5’) differentially expressed fragments in control (*n* = 4) and 2-weeks HFD (*n* = 4) groups (Tomar et al., 2024). (**h**) Relative abundance of tsRNAs (Asp-GTC, Ser-TGA-5’, Glu-CTC-5’) differentially expressed fragments in control (*n* = 3) and 3-months WD (*n* = 3) groups (Raad et al., 2021). (**i**) Gene ontology pathway analysis of the target genes of miR-486a and b (false discovery rate < 0.05). Data are presented as mean ± s.e.m. and were analyzed by unpaired two-tailed Student’s t-tests (f-h).

Here, we aimed to mimic the hyperactive state of AgRP neurons one piRNA that occur in obesity^17,18^. To achieve this, we employed Designer Receptors Exclusively Activated by Designer Drugs (DREADD), a well-known chemogenetic tool for selectively activating specific neuronal populations in the mouse brain^19^ .We bilaterally injected an excitatory DREADD adeno-associated viral (AAV-hM3Dq-mCherry) vector into the arcuate nucleus of the hypothalamus (ARH) in both *Agrp*^*Cre/+*^ and *Agrp*^*+/+*^ (Fig. 1a). Three weeks post-injection, we confirmed the restriction of AAV to AgRP neurons and validated its efficacy (Supplemental Fig. 1a-c). The DREADD ligand, Clozapine-N-Oxide (CNO), was administered to both *Agrp*^*Cre/+*^ and control *Agrp*^*+/+*^ mice in the absence of food. Four hours post-administration, mature sperm was harvested from the caudae epididymides, and RNAs were extracted from the sperm. The purity of sperm samples was validated by qPCR analysis of cell-type specific markers. This analysis showed strong enrichment for mature sperm markers such as *Protamine-2* (*Prm2*) and low enrichment for somatic and stem cell markers such as *E-cadherin* and *C-kit* (Supplemental Fig. 1d). These RNAs were then used to prepare a small non-coding RNA (sncRNA) library, which was subsequently sequenced (Fig. 1a).

After mapping reads to the reference genome and sncRNA annotation databases, we confirmed that the distribution and biotypes between the two groups were very similar (Fig. 1b). Sperm of control mice had microRNAs (miRNAs) at 32%, PIWI-interacting RNAs (piRNAs) at 14%, transfer RNA-derived small RNAs (tsRNAs) at 15% and ribosomal small RNAs (rsRNAs) at 25%. In comparison, sperm of AgRP neuron-activated mice showed miRNAs at 33%, piRNAs at 16%, tsRNAs at 12% and rsRNAs at 22%. This similarity suggests that the global sncRNA profiles of the two groups are largely comparable, indicating that broad changes in RNA expression or biotype distribution in sperm are not the primary effects of AgRP neuron activation on the sperm RNA payload.

Nevertheless, a closer examination revealed specific alterations in tsRNA fragment sizes upon AgRP neuron activation. We observed significant changes in tsRNA sizes of 30, 31 bp (*P* value < 0.05), and 32 bp (*P* value = 0.05), with a reduction of approximately 40% (Supplemental Fig. 2a). The rapid changes observed in tsRNA composition following AgRP neuron activation are particularly intriguing and could be explained by the dynamic nature of tsRNA biogenesis. tsRNAs are produced through enzymatic processing of mature tRNAs by specific enzymes, notably DNMT2 and angiogenin^10,20^. Additionally, single-cell analysis of the epididymis has revealed an abundance of nucleases capable of such RNA processing, highlighting the epididymis as a key site for rapid RNA modifications^21^. We hypothesize that AgRP neuron activation may influence these enzymatic processing pathways, providing a mechanistic explanation for the acute changes we observe in sperm tsRNA content. This mechanism would allow for rapid modifications in RNA fragments without requiring de novo RNA synthesis, consistent with the timeframe of our observations.

In contrast, miRNAs, piRNAs and rsRNAs did not show differences in the abundances of specific fragment sizes, suggesting that their processing and stability are not significantly affected by AgRP neuron activation (Supplemental Fig. 2b-d).

Building on these observations, we carried out a differential expression analysis to pinpoint sncRNAs with significant expression differences between the control and AgRP neuron-activated groups. Unexpectedly, this analysis revealed 195 sncRNAs that were upregulated and 45 sncRNAs that were downregulated in the sperm following AgRP neuron activation (*P*_*adj*_ < 0.05 and log_2_[fold change (FC)] > |1|), constituting approximately 2% of the total sncRNA pool (Fig. 1c). Although these changes represent only a small fraction of the sncRNA payload (2%), this is expected given the acute nature of the treatment. Among the affected biotypes, 26% were piRNA, 6% were rsRNA, 6% were tsRNA, and 2% were miRNA (Supplemental Fig. 2e). Additionally, a high fraction (60%) of the differentially expressed sncRNAs were without annotation, so our analysis focused on the annotated sequences to provide clearer insights into the specific sncRNAs influenced by AgRP neuron activation (Supplemental Fig. 2e). Among the differentially expressed sncRNAs that we detected, all tsRNAs, miRNAs, and rsRNAs were downregulated. Only piRNAs showed both upregulation and downregulation, indicating a more complex regulatory pattern for this biotype compared to the others (Fig. 1d).

To further contextualize our findings, we compared our data with three previous studies that analyzed the impact of short- and long-term obesogenic diets on sperm RNA payload composition^4,5,9^. Given the established role of AgRP neurons in regulating whole-body energy homeostasis, this comparison is particularly relevant. This intersection analysis revealed that the alterations in tsRNA-Ser-TGA-5’ and tsRNA-Glu-TTC-5’ are consistently observed across multiple studies (Fig. 1e). The recurrence of these specific tsRNAs across different experimental conditions underscores their potential role as key markers or mediators in the physiological responses to both AgRP neuron activation and dietary changes. This is particularly significant given the established role of tsRNAs in the epigenetic inheritance of metabolic traits, such as obesity^4,5,10,12^. Additionally, similar patterns of alterations for other tsRNAs, such as tsRNA-Asp-GTC and tsRNA-Glu-CTC-5’, reinforce the idea that specific tsRNAs are reliably affected by various physiological stimuli and dietary interventions, further linking neural activation and metabolic epigenetics (Fig. 1e). Importantly, the expression levels of tsRNAs Asp-GTC, Glu-CTC-5’, and Glu-TGA-5’ were significantly decreased in sperm following short-term high-fat diet (HFD)^5^, closely mirroring the downregulation observed after AgRP neuron activation (Fig. 1f-g). In contrast, long-term Western diet (WD)^9^ feeding resulted in a differential response: while tsRNAs Glu-CTC-5’ and Ser-TGA-5’ were decreased, tsRNA Asp-GTC was notably increased in sperm (Fig. 1h). We could not make this comparison with the long-term HFD study because the data from individual samples were not available^4^. These findings suggest that short-term dietary intervention and AgRP activation have a more similar impact on sperm tsRNA profiles, while long-term HFD produces distinct changes, indicating different underlying mechanisms at play. This unexpected regulation by AgRP neurons highlights a potential upstream regulatory link that influences the response of sperm tsRNAs to varying metabolic conditions.

For the other biotypes, we did not see such consistency. However, we identified miR-451a as significantly altered in both our data and the long-term HFD and WD studies, while miR-486 (a and b) was significantly altered in both our study and the short-term HFD and long-term WD studies. Given the more immediate relevance of short-term dietary changes to our AgRP activation model, we focused our Gene Ontology analysis on miR-486 (a and b) to understand the specific biological pathways they might influence. This analysis revealed that many pathways were related to biological regulation and developmental processes (Fig. 1i). This suggests that while the overlap in miRNA changes is less pronounced, the functional implications of these changes might still be significant, particularly in the context of early developmental stage. Finally, we only saw one piRNA that was significantly altered by a short period of HFD and AgRP activation (Fig. 1e). While our analysis examined individual piRNAs, future studies would benefit from analyzing piRNAs at the cluster level, as this approach better reflects their biological organization and function. Given the complex and still-emerging understanding of piRNA functions, it is difficult to draw definitive conclusions about their role and significance in the context of AgRP neuron activation and HFD feeding. Overall, this indicates that while tsRNAs show a clear and consistent response to both AgRP neuron activation and HFD feeding, the responses of other sncRNA biotypes may be more specific to the type of metabolic perturbation or involve different regulatory mechanisms.

Our findings should be considered in the context of the diverse pathways through which sperm RNA content can be modified. These range from direct effects on developing sperm cells in the testes to epigenetic modifications and RNA transfer via epididymosomes. The rapid changes we observe in sperm RNA content following AgRP neuron activation suggest that these neurons might influence these pathways through systemic signals or neural circuits that affect the reproductive tract.

Our findings should be considered in the context of the multiple pathways that modulate the sperm RNA payload. First, environmental and metabolic challenges exert direct effects on developing sperm cells in the testes, influencing spermatogenesis and the biogenesis of tRNA fragments^1,4,5^. Second, post-testicular processes critically shape the sperm RNA payload, as exemplified by the rapid modification mediated through the transfer of RNAs via epididymosomes, which facilitates active RNA exchange between epididymal cells and maturing sperm^11,12^. Finally, emerging evidence suggests that RNA stability and processing in the epididymis are tightly regulated by a variety of RNA-modifying enzymes. In addition to nucleases identified in single-cell analyses^21^, enzymes such as angiogenin^20^ and DNMT2^10^ have been implicated in the biogenesis and modulation of tsRNAs. This coordinated enzymatic control may enable a rapid reshaping of the sperm RNA landscape in response to external metabolic cues. The rapid changes we observe in sperm RNA content following AgRP neuron activation imply that these neurons might influence these pathways through systemic signals or neural circuits that affect the reproductive tract. This modulation could involve hormonal or autonomic mechanisms similar to those described in previous works addressing the neural control of reproductive functions^15,20^. Such mechanisms might enable a fast adjustment in the RNA processing machinery without requiring de novo RNA synthesis, thereby aligning with the acute time frame of our observations.

In summary, our study demonstrates for the first time that AgRP neuron activation significantly alters the sperm sncRNA payload, with notable downregulation of specific tsRNAs, miRNAs, and rsRNAs, while piRNAs exhibit both upregulation and downregulation patterns. The consistent downregulation of tsRNAs parallels earlier findings from studies on HFD and WD feeding. Our results indicate that the detrimental effects of obesity on sperm RNA composition may be partially mediated via a common upstream regulatory mechanism involving AgRP neurons. These results reveal a new and unexpected perspective on the modulation of epigenetic information carried by sperm mediated by hypothalamic AgRP neurons.

While our findings provide novel evidence that AgRP neurons can influence sperm RNA composition, we acknowledge that additional validation approaches would further strengthen these observations. Future work will be valuable to deepen our understanding of the molecular mechanisms connecting hypothalamic AgRP neuron activity to sperm RNA modifications, particularly focusing on the precise pathways mediating these rapid changes in RNA content.

## Methods

### Animals

The University of Barcelona Ethics Committee approved all animal research (Approval No. CEEA-390/19), which were conducted in accordance with the current laws in Catalonia, Spain, and Europe. Mice were housed in a controlled environment, maintaining a temperature range of 20–24ºC and a 12-hour light-dark cycle. The mice’s health condition was constantly evaluated. *Agrp*^*+/+*^ and *Agrp*^*Cre/+*^ were purchased from Jackson Laboratory (Strain #:012899). We crossed *Agrp*^*Cre/+*^ with *Agrp*^*+/+*^ to generate more *Agrp*^*+/+*^ with *Agrp*^*Cre/+*^. *Agrp*^*+/+*^ were used as control. All the methods and procedures were reported in accordance with ARRIVE guidelines.

### Stereotaxic surgery

Eight-week-old *Agrp*^*Cre/+*^ and *Agrp*^*+/+*^ male mice, were anesthetized using a Ketamine/Xylazine mixture (i.p., 100 mg/Kg and 10 mg/Kg) and secured in a stereotaxic frame (Kopf Instruments). For pain management and postoperative recovery, the mice were administered Buprenorphine (i.p., 0.3 mg/Kg) prior to surgery and every 12 h thereafter for 72 h. The surgical procedure began by exposing the skull and drilling a small hole for the precise injection of an adeno-associated virus (AAV) into the ARH. The AAV, containing excitatory Designer Receptors Exclusively Activated by Designer Drugs (DREADDs) encoding hSYN-DIO-hM3D(Gq)-mCherry (Addgene - # 44361-AAV9–1.10 × 10^13^ gc/mL), was delivered bilaterally (300 nL per side) using a 33-gauge needle attached to a 5 µL syringe (NeuroSyringe, Hamilton) at a rate of 50 nL/min. The coordinates used for the injections relative to Bregma were AP: -1.5 mm; ML: ± 0.3 mm; DV: -5.8 mm below the skull surface. After an 8-minute interval, the needle was raised 1 mm, held for an additional minute, and then fully retracted. The surgical site was sealed and the mice were kept on a heating pad at 37ºC until they fully recovered from the anesthesia, after which they were returned to their home cage.

### Chemogenetic activation of AgRP neurons

On experimental days, cage food was removed for 1 h. Then mice were intraperitoneally injected with Clozapine-N-oxide (CNO; 1 mg/kg body weight for activation; Tocris Bioscience - #4936) dissolved in sterile saline. Mice were euthanized 4-hours later. Correct injection sites were confirmed in every mouse post-mortem by assessing mCherry signal under fluorescent microscope (Nikon Eclipse Ni-U).

### Brain histology

Mice were perfused transcardially with ice-cold phosphate-buffered saline (PBS), followed by a fixative solution (4% PFA). Brains were dissected and post-fixed in 4% PFA at 4 °C for 24 h, then cryoprotected in 30% sucrose in 1x PBS (pH 7.4). Brain sections, 26 μm thick, were prepared using a cryostat (Leica Biosystems, IL, USA) at − 20 °C. For cFOS immunodetection, brain slices containing the ARH were extensively washed in PBS buffer and blocked in 3% BSA, 2% chicken serum in PBS plus 0.2% Triton X-100 for 1 h. Sections were incubated with a rabbit anti-cFOS antibody (1:500; Synaptic Systems - # 226008) in blocking buffer overnight at 4 °C. After washing with PBS 0.2% Triton X-100, slices were incubated with a chicken anti-rabbit Alexa Fluor 488 antibody (1:300) for 1 h at room temperature. After washing with PBS 0.2% Triton X-100, slices were mounted with Vectashield antifade mounting medium with DAPI (Vector Laboratories, USA). Images were taken using an Olympus BX41 fluorescence microscope equipped with a 20x objective.

### Mouse mature sperm extraction

Mature sperm extraction from mice was conducted according to the established protocol for murine in vitro fertilization. Initially, a Petri dish was set up with 1mL of 1x PBS in the middle, covered with paraffin oil (#76235 – Sigma). After euthanizing the male mouse via cervical dislocation, the caudae epididymides were removed and placed on the paraffin oil in the prepared dish. Each cauda was then cut using scissors at the midpoint to release the sperm clusters, which were gently moved toward the PBS in the center of the dish. The sperm remained in the PBS for 10 min at room temperature. Subsequently, the solution with the sperm was collected and spun at 600 g at 4ºC for 5 min. Finally, the sperm underwent two additional washes with 1x PBS.

### Small non-coding RNA sequencing (sncRNA-seq)

Mature sperm RNAs were extracted using Trizol (#15596026 – Thermo Fisher Scientific) following standard protocols. Quantification of each samples was performed using a Qubit fluorometer (Thermo Fisher Scientific). RNA integrity analysis was performed by analyzing each sample in a Agilent High Sensitivity RNA ScreenTape System (Agilent). Small non-coding RNA libraries were generated with 100ng of RNA with NEXTFLEX Small RNA-Seq kit v4 with UDIs (NOVA-5132-31 – Perkin Elmer). Libraries were sequenced on an Illumina NextSeq2000 (Illumina, Inc.) in single-end mode with a read length of 1 × 50 bp.

### Sperm purity assessment by qPCR

RNA was reverse transcribed using the High-Capacity cDNA Reverse Transcription Kit (#4368813, Thermo Fisher) following manufacturer’s instructions. qPCR was performed using TaqMan Gene Expression Master Mix (Applied Biosystems, #4369016) with TaqMan probes for *Prm1* (Mm01342731_g1, Thermo Fisher), *Prm2* (Mm03048199_m1, Thermo Fisher), *C-kit* (Mm00445212_m1, Thermo Fisher) and *E-cadherin* (Mm01247357_m1, Thermo Fisher) on a QuantStudio 5 Real-Time PCR System (Applied Biosystems). Relative gene expression was calculated using the 2^-ΔΔCt method with Prm1 as the reference gene.

### SncRNA-seq quality control and data analysis

After sequencing, we used the Small non-coding RNA annotation Pipeline Optimized for rRNA- and tRNA-Derived Small RNAs (SPORTS1.1)^22^ to trim, map and annotate fragments. We used SPORTS1.1 with default parameters in which reads were trimmed and filtered for low quality base-pairs. Sequencing duplicates were not removed. In addition, reads with a length shorter than 15 bp or greater than 45 bp were excluded from the analysis. Then, the remaining reads were mapped using bowtie^23^ to mouse reference genome and small RNA annotation databases, with no mismatches allowed. These were obtained from SPORTS1.1 as provided by the authors; including mm10 reference genome, miRNA from miRBase (release-21), rRNA curated sequences from National Center for Biotechnology Information (NCBI) Nucleotide, tRNA from GtRNAdb, piRNA from piRBase, other ncRNA from Ensembl (release-89), and Rfam (release-12.3).

SPORTS1.1 output files were used to generate normalized RPM counts for the different small RNA biotypes annotations per sample and fragment length. Small RNA biotype abundance per experimental group was calculated averaging the previous values.

Differentially expressed fragments analysis was carried out in R v4.3.1 with DESeq2 R package v1.40.2^24^ using raw fragment counts. Only sequences with counts > = 50 in at least 4 biological replicates were considered. Thresholds to define differentially expressed fragments (DEFs) were set using *P*_*adj*_ < 0.05 (using Benjamini-Hochberg FDR) and log2[fold change (FC)] > |1|. For a better estimation of log2(FC) we used shrunken log2(FC) with *apeglm* method^25^, which looks at the largest fold changes that are not due to low counts and uses these to inform a prior distribution, allowing to compare estimated log2(FC) across experiments. Heatmaps were made using VST-normalized expression values of DEFs using pheatmap R package v1.0.12.

Functional enrichment analysis with gene ontologies was conducted using g: GOSt from g: Profiler R package v 0.2.3 with predicted gene targets for miR-486; only significant functional pathways (Padj < 0.05) were considered. These targets were obtained from the TargetScanMouse database (v8)^26^, which contains information for predicted microRNA targets in mice. In this case, we extracted the predicted targets for the miR-486 conserved family.

We used the same approach and thresholds to reanalyze Tomar et al. mapped reads to obtain their set of DEFs. For Raad et al., we used DEFs as included in the study’s source data. Intersection between small RNA biotypes was restricted to annotations of each study’s DEFs, in which we evaluated whether their results included the same biotype annotations as in our analysis. Results were represented in a chord diagram made with the circlize R package v0.4.15^27^. We aggregated RPM values of each intersected tsRNA DEFs to compare their abundance between the respective experimental groups per study.

### Statistical analysis

Data is presented as the mean value accompanied by their respective standard errors (SEM). Statistical evaluations were conducted with GraphPad Prism software (version 10) or R v4.3.1. Details on the specific statistical tests applied to each study are indicated in the legends accompanying the figures.

## Electronic supplementary material

Below is the link to the electronic supplementary material.

Supplementary Material 1

Supplementary Material 2

## Data Availability

The sncRNA-Seq datasets generated and analyzed in this research can be found in the NCBI GEO repository with the accession number GSE277621.
