# Supplementary material for: AgRP neurons shape the sperm small RNA payload
Source: Sci Rep. 2025 Feb 28;15:7206. doi: 10.1038/s41598-025-91391-4 (PMC11871312; doi:10.1038/s41598-025-91391-4)
Supplement: Supplementary file 1 — Supplementary Material 1 [file 41598_2025_91391_MOESM1_ESM.pdf]

Supplemental Figure 1

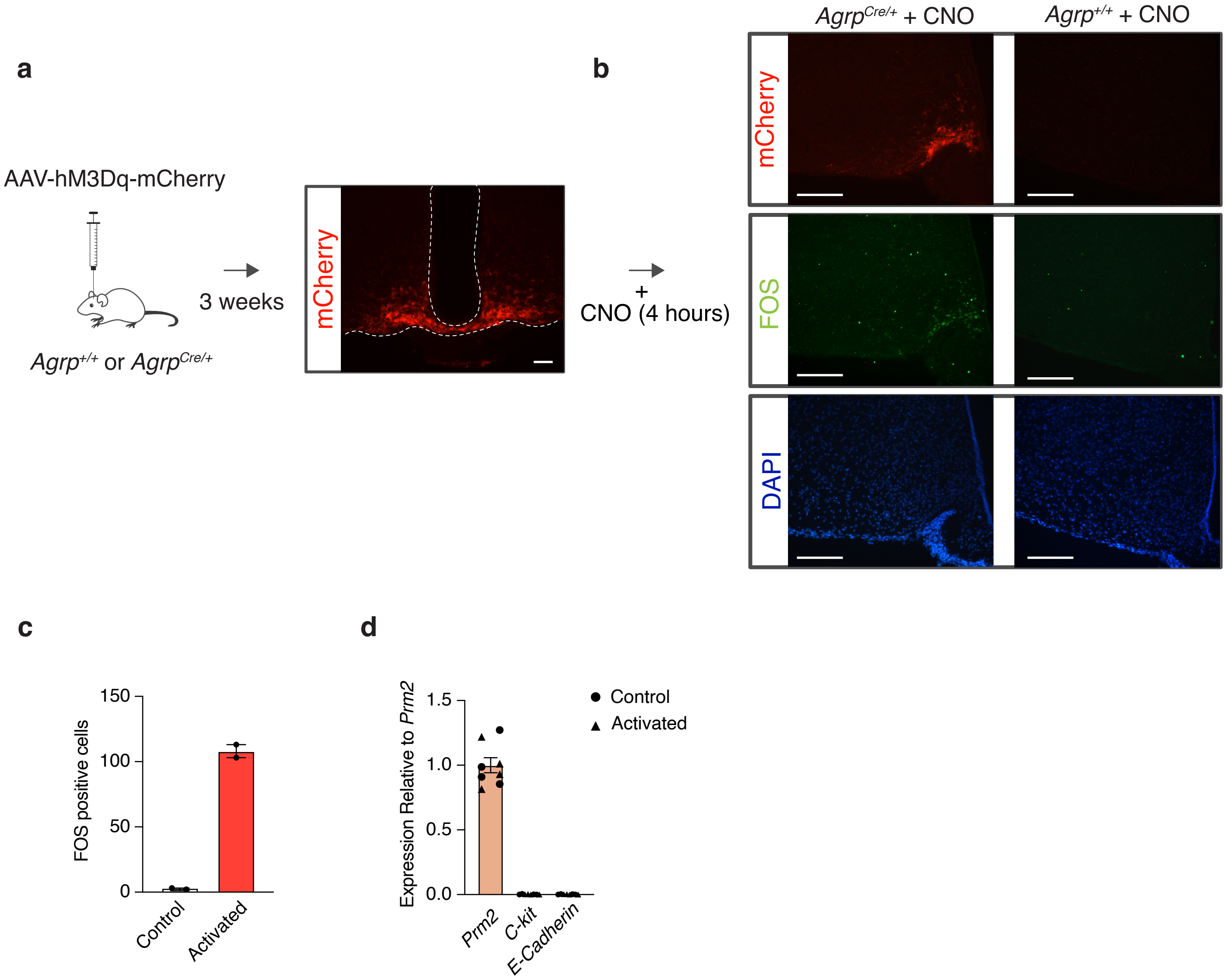

Supplemental Figure 2

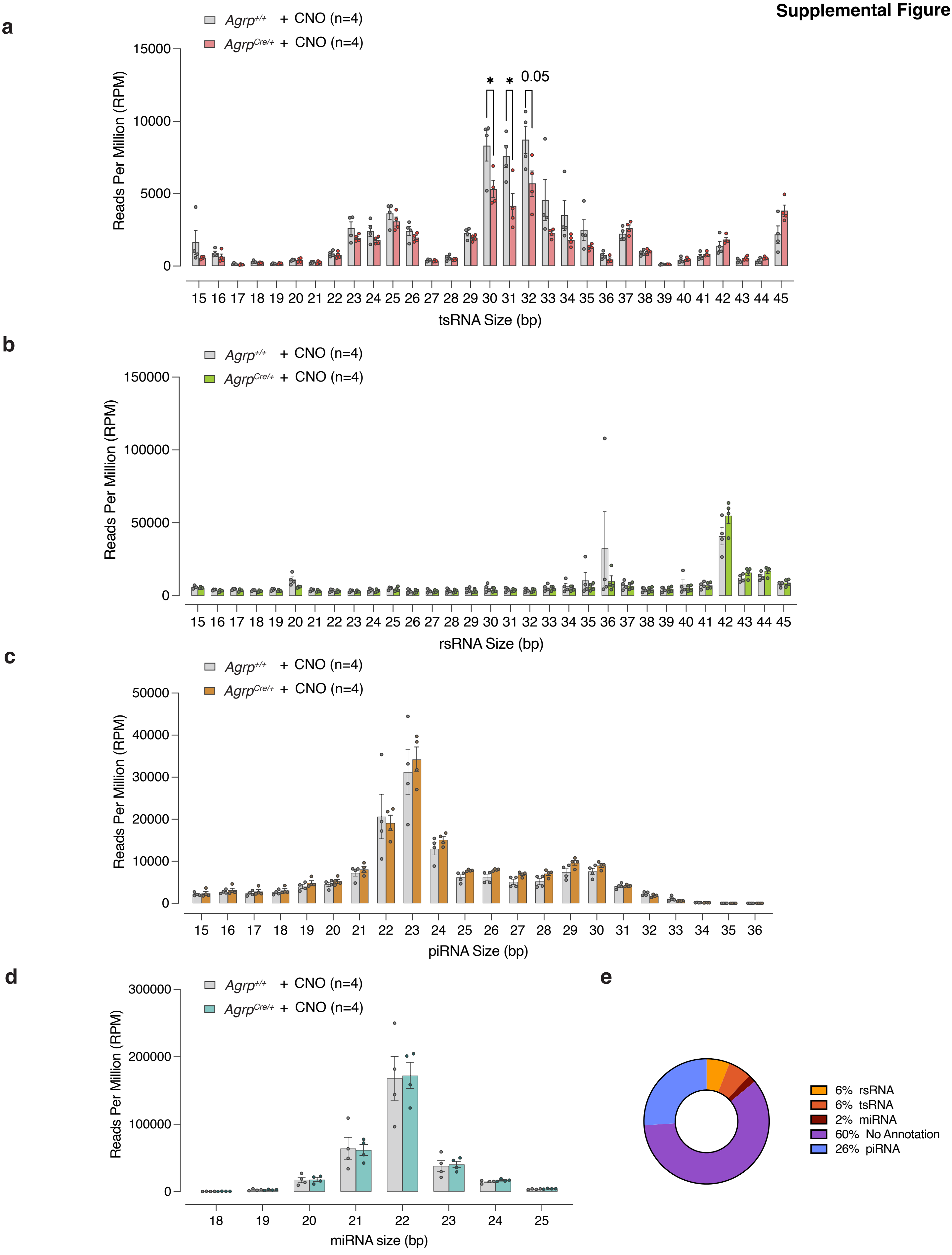

Supplemental Figure 1:

(a) Schematic of the approach to express the excitatory DREADD activator (hM3Dq) in AgRP neurons. The representative images show mCherry reporter expression in a coronal section containing the arcuate of the hypothalamus.

(b) Assessment of chemogenetic activation of AgRP neurons in $Agrp^{+/+}$ and $Agrp^{Cre/+}$ mice expressing DREADD activator (hM3Dq) 4 hours after CNO injection. Representative images of FOS (green), mCherry (red) and DAPI nuclei counterstaining (blue) are shown. Scale bar: 50µm. n = 2 per group.

(c) Quantification of FOS positive cells in $Agrp^{+/+}$ and $Agrp^{Cre/+}$ mice expressing DREADD activator (hM3Dq) 4 hours after CNO injection.

(d) Expression levels of *Prmt2*, *E-Cadherin*, and *C-Kit* measured by quantitative PCR (qPCR) in mature sperm from control (circle) or activated (triangle) mice (n=8).

Supplemental Figure 2:

Abundance of (a) tsRNA5', (b) rsRNA, (c) piRNA and (d) miRNA across different fragment size. Bp base pair.

(e) Percentage of the different biotypes that differentially expressed after AgRP activation.

Data are presented as mean ± s.e.m. and were analyzed by unpaired two-tailed Student's t-tests (a-d).
